# Biogeography and evolution of Asian Gesneriaceae based on updated taxonomy

**DOI:** 10.3897/phytokeys.157.34032

**Published:** 2020-08-26

**Authors:** Ke Tan, Tao Lu, Ming-Xun Ren

**Affiliations:** 1 Center for Terrestrial Biodiversity of the South China Sea, College of Ecology and Environment, Hainan University, Haikou 570228, China Hainan University Haikou China

**Keywords:** Didymocarpoideae, endemic, species diversification, limestone landscape, monsoon, long-distance dispersal

## Abstract

Based on an updated taxonomy of Gesneriaceae, the biogeography and evolution of the Asian Gesneriaceae are outlined and discussed. Most of the Asian Gesneriaceae belongs to Didymocarpoideae, except *Titanotrichum* was recently moved into Gesnerioideae. Most basal taxa of the Asian Gesneriaceae are found in the Indian subcontinent and Indo-China Peninsula, suggesting Didymocarpoideae might originate in these regions. Four species diversification centers were recognized, i.e. Sino-Vietnam regions, Malay Peninsula, North Borneo and Northwest Yunnan (Hengduan Mountains). The first three regions are dominated by limestone landscapes, while the Northwest Yunnan is well-known for its numerous deep gorges and high mountains. The places with at least 25% species are neoendemics (newly evolved and narrowly endemic) which were determined as evolutionary hotspots, including Hengduan Mountains, boundary areas of Yunnan-Guizhou-Guangxi in Southwest China, North Borneo, Pahang and Terengganu in Malay Peninsula, and mountainous areas in North Thailand, North Sulawesi Island. Finally, the underlying mechanisms for biogeographical patterns and species diversification of the Asian Gesneriaceae are discussed.

## Introduction

Gesneriaceae Rich. & Juss. ex. DC. is a middle-sized family, including about 150 genera and over 3400 species (Weber et al. 2013). Traditionally, the family was divided into two subfamilies, subfamily *Cyrtandroideae* Burnett (palaeotropical group, with superior ovary and two unequal cotyledons) and subfamily *Gesnerioideae* Burnett (neotropical group, with inferior ovary and two equal cotyledons) (Burtt 1998).

Based on recent molecular and morphological data, Weber et al. (2013) put forward a new classification system, consisting of three subfamilies: *Sanangoideae* Weber (monotypic genus, endemic to South America), *Didymocarpoideae* Arn. and *Gesnerioideae* Burnett. In this newest classification system, the monotype genus *Titanotrichum* Solereder in Asia (China and Japan) has been transferred to the subfamily Gesnerioideae, which was formerly treated as “New World Gesneriaceae” (Burtt 1998; Weber et al. 2013). However, Didymocarpoideae is still “Old World Gesneriaceae”, consisting of 67 genera and more than 2300 species (Möller et al. 2017, Xu et al. 2017).

Recently, many Asian Gesneriaceae taxa had experienced extensive revision for their systematic positions, such as *Boea* Commerson ex Lamarck (Puglisi and Middleton 2018), *Microchirita* (C.B.Clarke) Y.Z. Wang (Middleton 2018), *Henckelia* Spreng. (Middleton et al. 2013), *Paraboea* (C. B. Clarke) Ridley (Puglisi et al. 2016) and the enlarged genus *Oreocharis* Bentham (Möller et al. 2011, 2014; Chen et al. 2014). Furthermore, several genera were newly established, i.e. *Billolivia* D.J. Middleton (Middleton et al. 2014), *Chayamaritia* D.J. Middleton (Middleton et al. 2015), *Middletonia* D.J. Middleton (Puglisi et al. 2016; Puglisi and Middleton 2017), *Rachunia* D.J. Middleton (Middleton et al. 2018). Such significant revisions of so many genera call for an updated study about biogeography and evolution of the Asian Gesneriaceae.

In this paper, we collected the species locality data (coordinates) from GBIF (Global Biodiversity Information Facility, https://www.gbif.org/) for all the species of the Asian Gesneraiceae. The species diversity and systematic positions of all genera were determined according to the newest literatures (e.g. Möller et al. 2016a, b, 2017; Roalson and Roberts 2016; Puglisi and Middleton 2018). We used the software DIVA-GIS 7.5 to create a distribution map at 1° × 1° latitude/longitude grid resolution to reveal distribution patterns of species diversity and endemism. We also analyzed the distribution type of all the genera and identified evolutionary hotspots, i.e. the center of neoendemic species, which is determined when at least 25% of total species are locally endemic. Finally, we discussed the possible mechanisms, including both intrinsic and extrinsic factors, to explain the formation and maintenance of diversification and endemic centers of the Asian Gesneriaceae.

## 1 Distribution type

Based on Wu’s (1979, 1991) criterions and Li’s (1996) pioneer study on the geographical areal-types of the Asian Gesneriaceae, we identified the Asian Gesneriaceae as belonging to three areal types and 20 subtypes as below.

I. Pantropics

I1. Tropic Asia and tropic America disjuncted: *Rhynchoglossum* Blume.

I2. Tropic Asia to tropic Australia: *Boea* Comm. ex Lam., *Cyrtandra* J. R. Forster & G. Forster, *Stauranthera* Bentham, *Rhynchotechum* Blume.

I3. Tropic Asia to tropic Africa: *Epithema* Blume.

II. Tropical and subtropical Asia

II1. Widespread in tropical and subtropical Asia: *Aeschynanthus* Jack, *Paraboea* (C. B. Clarke) Ridley, *Dorcoceras* Bunge, *Codonoboea* Ridl., *Didymostigma* W. T. Wang, *Henckelia* Spreng., *Didymocarpus* Wallich.

II2. East India to Java: *Boeica* C. B. Clarke, *Leptoboea* Benth., *Beccarinda* Bentham, *Microchirita*, *Middletonia* D.J. Middleton.

II3. Indo-China Peninsula: *Tetraphyllum* C.B.Clarke, *Deinostigma* W.T.Wang & Z.Y.Li, *Damrongia* Kerr ex Craib, *Kaisupeea* B.L. Burtt, *Tribounia* D.J. Middleton ex M. Möller, *Billolivia*, *Chayamaritia*, *Rachunia* D.J. Middleton.

II4. North of Indo-China Peninsula: *Pseudochirita* W. T. Wang, *Anna* Pellegrin.

II5. Subtropic Asia (Southwest and South China): *Primulina* Hance, *Whytockia* W. W. Smith, *Hemiboea* C. B. Clarke, *Glabrella* M. Möller & W. H. Chen, *Gyrocheilos* W. T. Wang, *Raphiocarpus* Chun, *Petrocodon* Hance, *Allostigma* W. T. Wang, *Allocheilos* W. T. Wang, *Gyrogyne* W. T. Wang, *Litostigma* Y.G. Wei, Fang Wen & M. Möller.

II6. Malay Peninsula to Southwest China: *Ornithoboea* Parish ex C. B. Clarke.

II7. Hainan Island: *Cathayanthe* W. Y. Chun, *Metapetrocosmea* W. T. Wang.

II8. Sri Lanka and India: *Championia* Gardn., *Jerdonia* Wight.

II9. Malay Peninsula: *Orchadocarpa* Ridl., *Emarhendia* R. Kiew, A. Weber & B.L. Burtt, *Senyumia* R. Kiew, A. Weber & B.L. Burtt, *Somrania* D.J. Middleton, *Spelaeanthus* R. Kiew, A. Weber & B.L. Burtt.

II10. Malay Archipelago: *Agalmyla* Blume, *Monophyllaea* R. Br., *Loxocarpus* R. Br., *Loxonia* Jack, *Didissandra* C.B. Clarke, *Liebigia*, *Ridleyandra* A. Weber & B.L. Burtt.

II11. Borneo Island: *Hexatheca* C.B. Clarke.

III. North Temperate

III1. Widespread in East Asia: *Oreocharis* Bentham.

III2. Sino-Himalaya: *Corallodiscus* Batalin, *Loxostigma* C.B. Clarke.

III3. Sino-Japan: *Conandron* Sieb. & Zucc., *Titanotrichum* Solereder.

III4. Hengduan Mountains to Yunnan Plateau: *Rhabdothamnopsis* Hemsl.

III5. Hengduan Mountains to Central China: *Briggsiopsis* K. Y. Pan, *Petrocosmea* Oliver.

III6. Himalaya: *Platystemma* Wallich.

## 2 Geographical distribution patterns

**Figure 1.**
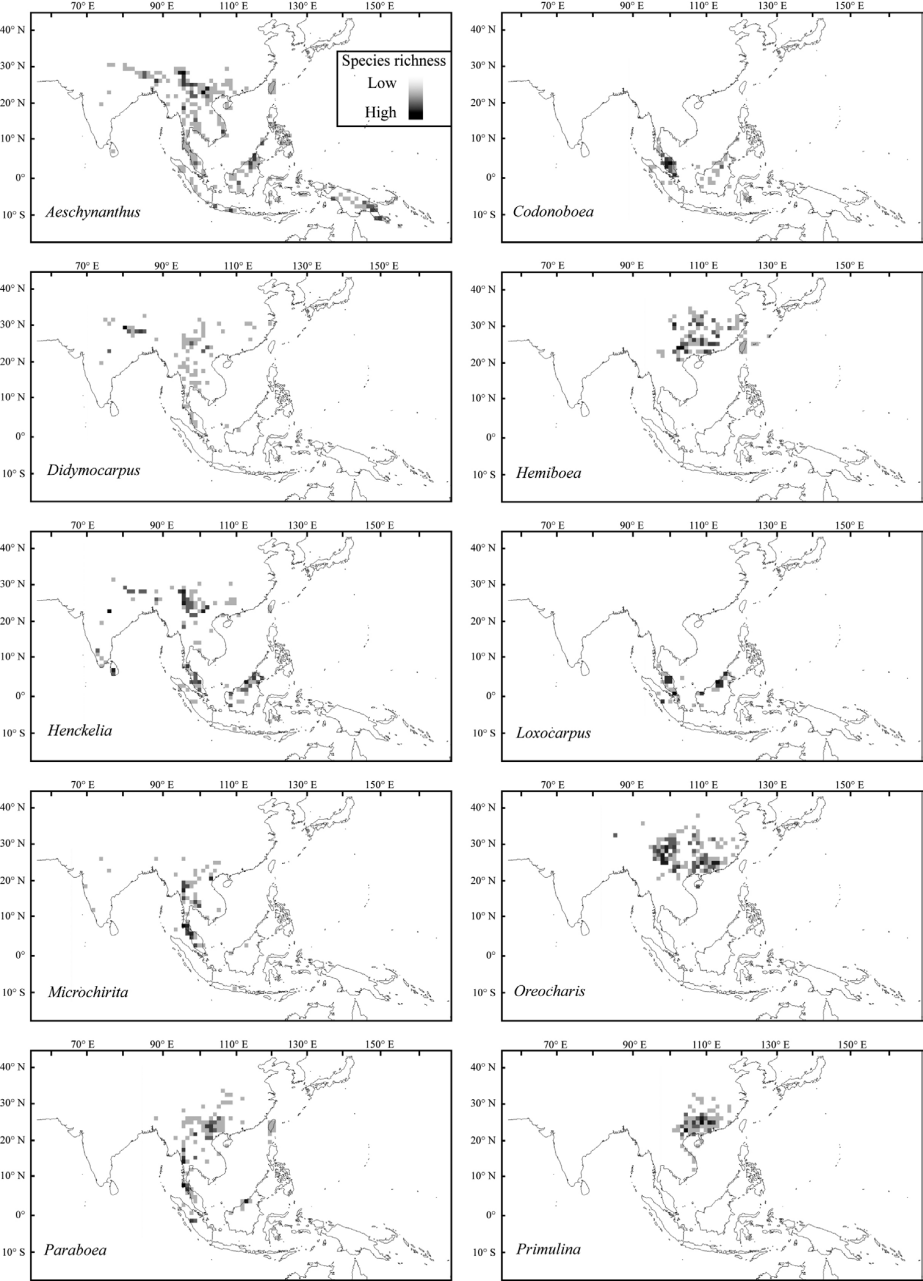
Geographical distribution patterns of the 10 genera of the Asian Gesneriaceae that experienced extensive changes in species compositions.

**Figure 2.**
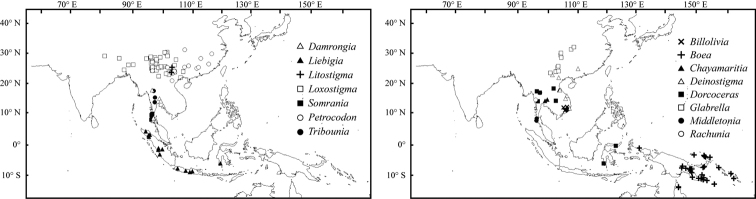
Distribution localities of 15 genera of the Asian Gesneriaceae that experienced extensive changes in species compositions.

**Figure 3.**
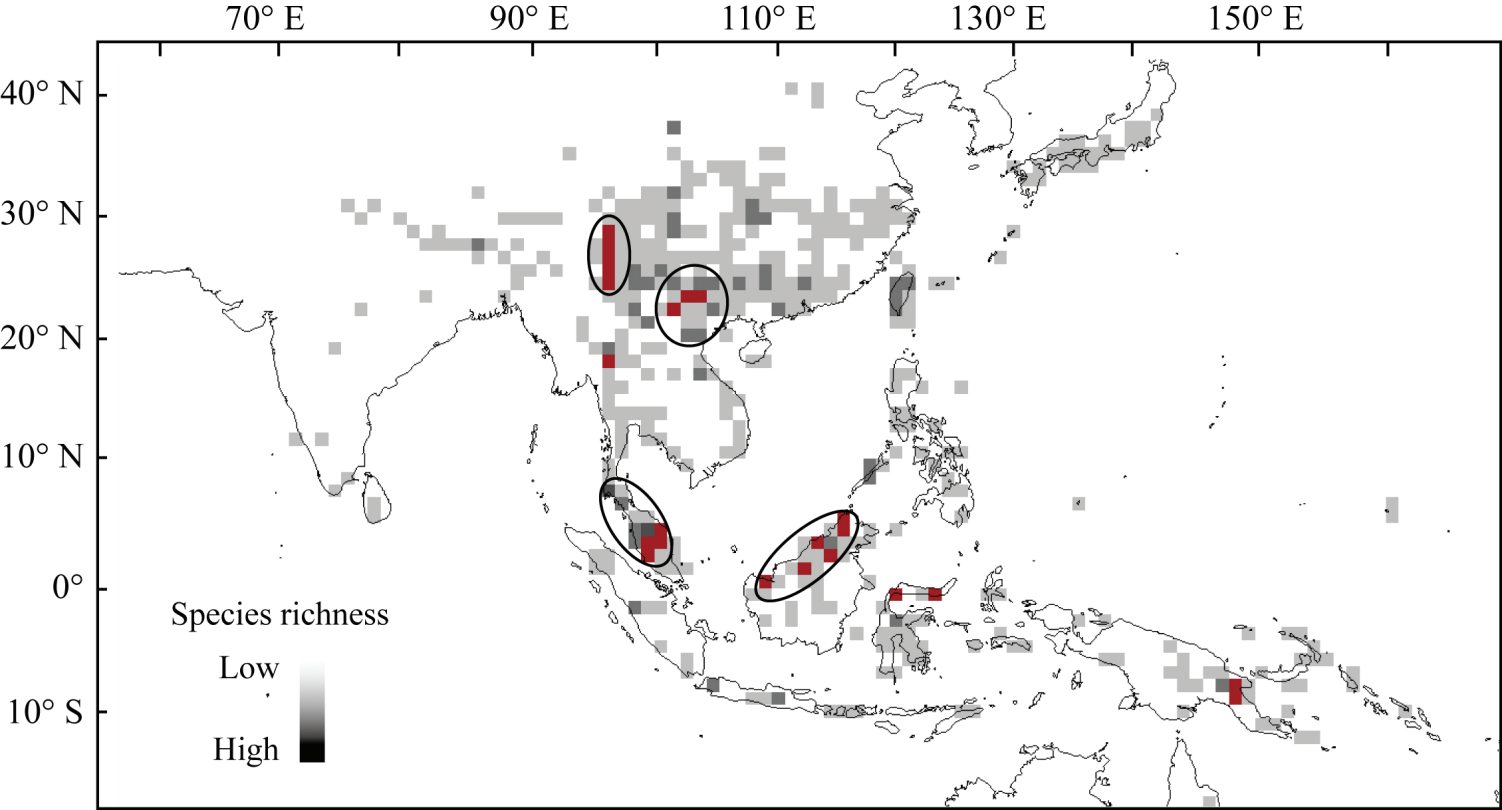
Species distributions pattern of the Asian Gesneriaceae. Black circles indicate diversification centers with highest species richness and the red grids are the evolutionary hotspots (at least 25% species are neoendemics). The species distribution information is obtained from http://www.gbif.org. The map was drawn using DIVA-GIS7.5.

Tropical and subtropical Asia are the distribution centers of the subfamily Didymocarpoideae, harbouring 85% genus and more than 90% species of Didymocarpoideae. Indo-China Peninsula and Southwest China (Figs 1, 2), which are dominated by limestone landscapes (Clements et al. 2006), are places notable for recording the highest species density (Fig. 1 and Fig. 3). According to the updated phylogeny of the Asian Gesneriaceae (Möller et al. 2016b, 2017; Xu et al. 2017), most basal taxa of the Didymocarpoideae, like *Rhynchotechum* and *Corallodiscus* (Fig. 3), occur at India and Indo-China Peninsula and the nearby regions such as Sino-Vietnam region and Southwest Yunnan. This suggests that modern Didymocarpoideae probably originated in these regions.

### 2.1 Diversification and endemic centers

Our data recognized four species diversification centers (places with highest values of species density), i.e. Sino-Vietnam Region including boundary areas of Guizhou-Yunnan-Guangxi in Southwest China, Northwest Yunnan (Hengduan Mountains), Malay Peninsula and North Borneo (Fig. 3). In a study focusing on China’s Gesneriaceae, Liu et al. (2017) found that richness of Gesneriaceae peaked in Southwest China, and Hengduan Mountains and boundary areas of Guizhou-Yunnan-Guangxi are the most significant hotspots of species diversity and endemism. Our results closely coincided with their findings.

**Figure 4.**
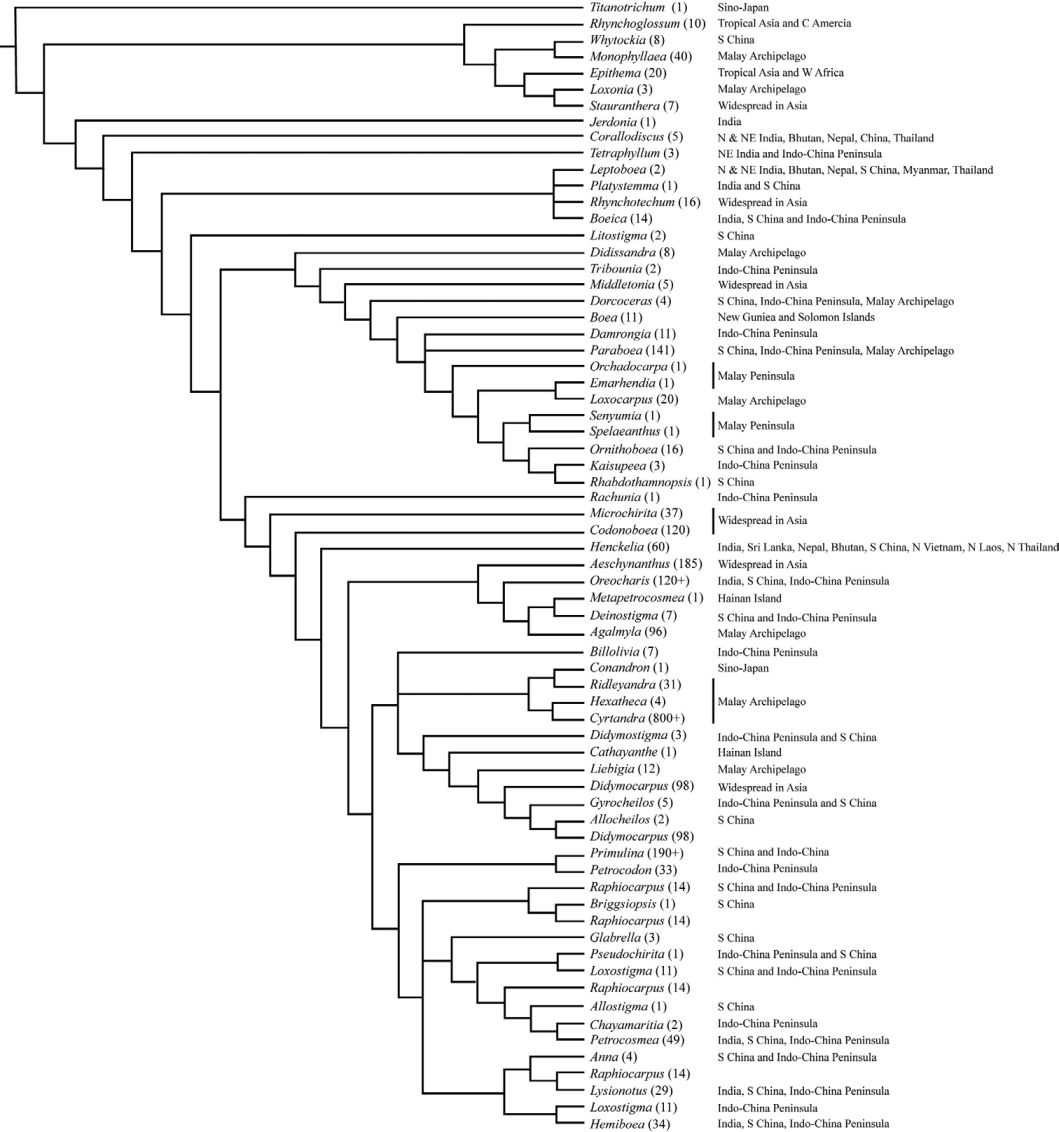
Genera phylogeny with geographical distribution pattern of the Asian Gesneriaceae. The number in the brackets is the species diversity of the genus. Phylogeny tree was redrawn based on Möller and Clark (2013), Middleton et al. (2015), Puglisi et al. (2016), Möller et al. (2016a), Middleton et al. (2018).

Indo-China Peninsula turned out to be an extraordinary diversification center, harbouring several endemic genera *Tribounia*, *Billolivia*, *Chayamaritia* (Fig. 2). These genera are newly evolved and contain very few species (Fig. 4). The Indian subcontinent has the lowest value of species density, with only two endemic genera, i.e. *Jerdonia* and *Championia* (Sri Lanka). *Boea* was no longer widespread in tropic Asia and its endemic center appeared in Papua New Guinea and the Solomon Islands (Puglisi et al. 2016; Puglisi and Middleton 2018) (Fig. 2). Based on molecular data from Möller and Clark (2013) and Roalson and Roberts (2016), most locally endemic species such as in *Aeschynanthus* and *Cyrtandra* were newly evolved, i.e. neoendemics.

### 2.2 Evolutionary hotspots

We determined places where at least 25% species are local endemics as ‘evolutionary hotspots’. Six evolutionary hotspots were identified, i.e. Northwest Yunnan (Hengduan Mountains), boundary areas of Yunnan-Guizhou-Guangxi in Southwest China, mountains in North Thailand, Malay Peninsula, North Borneo, and North Sulawesi (Fig. 3). These evolutionary hotspots were distributed largely in the diversification centers (Fig. 3), similar to findings of Liu et al. (2017) in which Hengduan Mountains and boundary areas of Yunnan-Guizhou-Guangxi were recognized as two hotspots of species richness of Chinese Gesneriaceae.

The geography of these six evolutionary hotspots was the most complex area in tectonic history, formed by the interaction of Indian, Eurasian, Australian and Pacific Plates (Hall and Spakman 2015). Therefore, the highly fragmented islands and limestone landscapes in Southeast Asia probably facilitated speciation of the Asian Gesneriaceae, similar pattern found in *Begonia* (Chung et al. 2014) and *Alocasia* (Nauheimer et al. 2012).

## 3 Origin and Evolution of the Asian Gesneriaceae

Burtt (1998) proposed that Gesneriaceae is of southern hemisphere (Gondwana) origin, with the Gondwana broken down and dispersed all over the world. This hypothesis is based on Gesnerioideae spreading to South America via the Antarctic and Didymocarpoideae by migrating northwards, ‘dropping’ representatives in Africa and Madagascar and finally reaching the Eurasiatic continent and spreading from there to the Malay Archipelago and the Pacific. This hypothesis, however, faces difficulties both from the geological time scale and the molecular data. Perret et al. (2013) reconstructed the biogeography history and suggested an origin of this family in southern America during the late Cretaceous period. The Gondwana break-up, however, began at about 150 Ma (Hall and Spakman 2015). Woo et al. (2011) indicated that there were two independent long-distance dispersals or overland migrations from South America to Australasia via Antarctica, but how they entered Asia or Africa is still unclear. The molecular data from Möller et al. (2009) and Roalson and Roberts (2016) show that the most basal species were found on the Indian subcontinent, such as *Jerdonia* (mountains of SW India), *Corallodiscus* (Himalayas and China), *Tetraphyllum*, *Leptoboea* and *Boeica* (Himalayas and adjacent areas). Only *Rhynchotechum*, about 18 species, which is widespread from Himalayas to Malay Archipelago, and one other species, even reaches New Guinea. Furthermore, *Boea* was the only endemic genus at the east side of the Huxley’s Line. Therefore, the Asian Gesneriaceae may have originated from the Indian subcontinent and/or Indo-China Peninsula, then dispersed to the east and the north and finally reached Southeast Asia and East Asia.

An up-to-date phylogeny indicated that Didymocarpoideae and Gesnerioideae probably separated at about 74 Ma (Roalson and Roberts 2016), when the Indian Plate had been separated from Gondwana (Hall and Spakman 2015). We propose two hypotheses for the origin of Didymocarpoideae. One is in India, whereby the ancestor of Didymocarpoideae dispersed via the south of South America and Antarctica to India, until about 45 Ma when the Indian Plate collided with the Eurasia Plate and rapid speciation occurred. This is in accordance with Burtt’s (1998) “Out of India” hypothesis.

The new position of *Titanotrichum* in the Gesnerioideae (Wang et al. 2004) suggests a possible dispersal event from the New World to Asia. Perret et al. (2013) proposed that the ancestor of *Titanotrichum* might either disperse across Beringia from North America to East Asia or originate at East Asia. This genus is distinctive for small and numerous bulbils in the inflorescence, which evolved at about Miocene (Wang and Cronk 2003) and probably facilitated its long-distance dispersal.

The alternative hypothesis is that the Asian Gesneriaceae might follow the ‘Malpighiaceae Route’ via the ‘North Atlantic land bridge’ to Eurasia (Davis et al. 2002). However, currently very limited experimental studies focusing on this scenario have been carried out and we cannot speculate on details about this hypothesis. To test such a hypothesis, molecular biogeographic studies on a pantropical genus such as *Epithema and Rhynchoglossum* are needed to figure out their historical dispersal route(s) between tropical America, Africa and Asia.

## 4 Mechanisms for species diversification

### 4.1 Growth forms

According to Roalson and Roberts (2016), epiphytism and unifoliate growth are two important growth forms impacting on the diversification rate in Gesneriaceae, like *Monophyllaea* and a section of *Streptocarpus* Lindl. in the Old World. Epiphytes and non-epiphytes have the same speciation rate, but epiphytism has a much lower extinction rate (Roalson and Roberts 2016). This means that epiphytism *per se* is not the driving factor for speciation, it probably can lower the extinction rate or is associated with other traits that promoting speciation. For example, there is a strong correlation between the epiphytic habit and ornithocory (dispersal of seeds by birds) (Weber 2004). In orchids, epiphytism is also often correlated with CAM photosynthesis (Givnish et al. 2015), but in Gesneriaceae only the New World *Codonanthe crassifolia* was confirmed to have some CAM-like characteristics (Guralnick et al. 1986).

Unifoliate growth form strongly suggests that growth form positively influences speciation rate (Roalson and Roberts 2016). Previous work on *Streptocarpus* shows the evolution of growth forms, especially rosulate and unifoliate growth and they believed this was an adaptation for deep shade and unifoliate growth increasing diversification rate (Möller and Cronk 2001). *Cyrtandra* has, however, also undergone a significant increase in speciation rate, probably associated with other characteristics such as seeds’ dispersal mode.

### 4.2 Specialized pollination adaptations

Normally, most Gesneriaceae species exhibiting zygomorphic flowers are thought to be more specialised in pollination adaptation, since it restricts pollinator behaviour and can therefore increase pollination efficiency (Gong and Huang 2009; Ling et al. 2017). Wang et al. (2010) and Ling et al. (2017) proposed that actinomorphy probably is a derived trait in the Asian Gesneriaceae, which is associated with shifts in pollination strategies, such as nectar- to pollen-rewards and/or from specialist to generalist pollinators.

Most Asian Gesneriaceae has the fused anthers, normally anthers are united by pairs, such as *Loxostigma* and *Anna* (Weber 2004). In some species, all the four anthers are fused or adnated, such as *Beccarinda* and *Stauranthera* (Weber 2004). Anther fusion can assemble the anthers to the same position and facilitate all the anthers touching the same locality of the pollinator’s body, which can greatly enhance the precision of cross-pollination (Ren 2008; Ren and Tang 2010). Wang (1990) indicated that anthers fusion with all stamens in the flower is late evolved than anther fusion in pairs and show a relatively higher level of pollination efficiency and consequently probably facilitate species diversifications.

There is a highly specialized pollination mechanism in the Asian Gesneriaceae, i.e. mirror-image flowers (Gao et al. 2006; Lu et al. 2019). Mirror-image flowers can be found in five genera in the Asian Gesneriaceae, i.e. *Paraboea* (Gao et al. 2006), *Ornithoboea*, *Didymocarpus*, *Rhabdothamnopsis*, and *Henckelia* (Lu et al. 2019). Mirror-image flowers are a sexual polymorphism in which the style deﬂects either to the left or the right side of the ﬂoral axis (Jesson and Barrett 2002; Gao et al. 2006). Jesson and Barrett (2002, 2003) pointed out that mirror-image flowers can increase the precision of cross-pollen transfer and may play an important role in pollination isolation and speciation. Normally, mirror-image flowers have reciprocal deflecting stamen(s) to the other side as compared with the deflecting style (reciprocal mirror-image flowers), which greatly increases pollen transfer efficiency (Jesson and Barrett 2003; Ren et al. 2013), but most mirror-image flowers in the Asian Gesneriaceae are non-reciprocal, without a deflecting stamen (Lu et al. 2019). Such unusual floral syndromes indicate unusual pollination adaptations in these species, but are awaiting further study.

### 4.3 Fruit adaptations to long-distance dispersal

In angiosperms, fruits significantly increase adaptive ability to withstand harsh environments and facilitate seed dispersal (Weber 2004). Fruit trait probably is a key trait for the high speciation rate and widespread range of both *Aeschynanthus* and *Cyrtandra* (Roalson and Roberts 2016). The hair-like appendages of seed in *Aeschynanthus* provide a favourable surface area to mass ratio (Denduangboripant et al. 2001), adapting to wind dispersal.

In *Cyrtandra* and *Rhynchotechum*, soft-fleshy (a true berry) fruits facilitate their colonisation throughout the Southeast Asia islands and nearby Pacific islands via bird dispersals (Cronk et al. 2005; Liu et al. 2017), and numerous islands in this area then promoted the speciation of the genus.

### 4.4 Extrinsic (environmental) factors

Compared to other regions, Asia is mostly dominated by the monsoon climate. There are three main types of summer monsoons in Asia, i.e. East Asia Monsoon, South Asia Monsoon and North-west Pacific Ocean Monsoon (Jiang et al. 2017; Kong et al. 2017). These three monsoons interact at Southwest China and Indo-China Peninsula and bring a large quantity of warm and wet air, thus providing a precondition for tropical plants to exist and facilitate the long-distance dispersal of the propagules. Monsoons do not only facilitate the northwards spread of tropical plants, but also aggravate the isolation of local habitat via the alternate dry and rainy seasons (Jiang et al. 2017), which might be related with the formation of diversification centers and evolutionary hotspots in Southwest China and Sino-Vietnam regions (Wang et al. 2011). More specifically, temperature changes since the Last Glacial Maximum had stronger effects on richness of rare species (Kong et al. 2017; Liu et al. 2017) while richness of common species was determined largely by current temperature seasonality such as monsoon climate (Liu et al. 2017).

Many studies had pointed out that microhabitat isolation caused by various landscapes such as limestone is the main factor for speciation in the Asian Gesneriaceae (Wang 1990; Ren 2015; Liu et al. 2017; Shui and Chen 2017). Especially for the three diversification and endemic centers, i.e. Sino-Vietnam Region, Malay Peninsula, North Borneo, the local landscapes are characterised by the various types of limestone landscapes (Clements et al. 2006; de Bruyn et al. 2014). Northern Borneo and Malaysia Peninsular were separated by the South China Sea, but they were connected during the glacial period and acted as a “land bridge” for plant dispersal across Southeast Asia (Hall and Spakman 2015). Frequent alternation of transformation between sea and continent intensified speciation in this area (de Bruyn et al. 2014).

Southwest China has the largest continuous limestone areas in the world, which includes Guizhou, Yunnan, Guangxi provinces and the Sino-Vietnam region (Clements et al. 2006; Hou et al. 2010; Ren 2015; Kong et al. 2017). South Yunnan is not only rich in limestone landscapes, but also comprises a series of spectacular north-south trending ridges along three major rivers of Asia: the Salween, Mekong and Red River, which have formed many unique microhabitats and microclimates, such as dry and hot valleys in Yunnan (Jiang et al. 2017). These diverse landscapes, forming ‘microhabitat islands’, greatly facilitated plant speciation (Clements et al. 2006; Ren 2015). Clements et al. (2006) and Chung et al. (2014) also proposed that these kinds of limestone microhabitats were formed largely by the East Asian monsoon (Jiang et al. 2017).

Northwest Yunnan (Hengduan Mountains), located at the eastern fringe of the Tibetan Plateau, is widely recognised as a globally important biodiversity hotspot (Myers et al. 2000; Liu et al. 2017) and the cradle of new species with an extraordinarily high ratio of recently evolved endemic species (neoendemics) that resulted from the uplift of the Himalayas and surrounding mountains (López-Pujol et al. 2011). It is noteworthy that not only neoendemics of Gesneriaceae have been found here, but most of the basal taxa also appeared here (Möller et al. 2009; Möller and Clark 2013; Roalson and Roberts 2016). Such a distribution pattern may relate to the Asian Gesneriaceae migration route (Möller et al. 2009).

In conclusion, we have discussed the biogeographic and diversification patterns of the Asian Gesneriaceae, along with underlying mechanisms of the family’s dispersal, adaptation and evolution. The family is still undergoing quick diversification and is awaiting further detailed studies not only about ecological adaptations but also evo-devo examinations on relationships between micro- and macro-evolution. Molecular biogeographic studies on the typical pantropic taxa using updated techniques such as sequenced restriction-site associated DNA (Baird et al. 2008; Feng et al. 2017) are also suggested to explore the historical dispersal patterns and evolutionary diversification of the family from tropical America to Africa and Asia.

**Table 1.** List of present genera of Asian Gesneriaceae.

Gernus	Distribution	Habitat	Species number	Taxonomic status	Reference
**Subfamily Gesnerioideae**					
**Tribe Titanotricheae**					
* Titanotrichum *	E China (Fujian and Taiwan) and Japan	Shaded areas in valleys; altitude 100–1200 m.	1	Placed in subfam. Gesnerioideae	Perret et al. 2013; Weber et al. 2013
**Subfamily Didymocarpoideae**					
**Tribe Epithemateae**					
* Epithema *	Central tropical Africa, India, Sri Lanka, Nepal, southern China and through Southeast Asia and Malesia to the Solomon Islands.	Shaded limestone rocks or caves in valleys.	20	No change at genus level	Bransgrove and Middleton 2015
* Gyrogyne *	S China (Bose Xian, W Guangxi)	Shaded waysides in hilly regions at low elevations.	1	Position in Epithemateae-Loxoniinae uncertain	
* Loxonia *	Southeast Asia (Sumatra, Malay Peninsula, Borneo, Java)	Damp places and humid rocks in deep shade.	3	No change	
* Monophyllaea *	Throughout Sumatra to New Guinea and from S Thailand and Luzon to Java	Limestone rocks, in shady forests, at cave entrances and below rocks.	>40	No change	Möller et al. 2016b
* Rhynchoglossum *	From India and S China to New Guinea, but one species distributed in Central America (Mexico, Colombia, Venezuela)	Wet and shady (preferably limestone) rocks, in forest or open, shady places; usually in the lowlands.	16	No change	
* Stauranthera *	from NE India and S China throughout Malaysia to New Guinea	Wet rocks and damp places in lowland rain forest.	7	No change	
* Whytockia *	S China (Sichuan, Guangxi, Hunan, Hubei, Guizhou, Yunnan and Taiwan)	Shaded and moist areas in valleys, shaded streamside rocks and stream banks, altitude 500–2200 m.	8	No change	
**Tribe Trichosporeae**					
* Aeschynanthus *	From S China, N & S India throughout Malesia to New Guinea and the Solomon Islands	Epiphytically on trees (rarely on rocks or bare soil), lowland or montane rain forest.	~185	Emended by inclusion of *Micraeschynanthus*	
* Agalmyla *	Malaysia (Sumatra, Malay Peninsula, Borneo, Java, Sulawesi, New Guinea)	Lowland and montane rainforest, mostly climbers.	96	No change	
* Allocheilos *	S China (Guizhou, E Yunnan)	Rocks in limestone hills, altitude ca. 1400 m.	2	No change	
* Allostigma *	S China	Limestone pavements; altitude ca. 200 m.	1	No change	
* Anna *	China, N Vietnam	Grassy slopes or forests, rock crevices in limestone hills.	4	No change	
* Beccarinda *	NE India, Burma, S China, Vietnam, Sumatra	Probably growing on humid rocks.	8	No change	
* Billolivia *	E Vietnam (Lam Dong)	Submontane tropical evergreen closed forest at 1550 m alt.	7	Re-established for five species of *Cyrtandra*	Middleton et al. 2014
* Boea *	Eastern Indonesia, Papua New Guinea, the Solomon Islands and Queensland (Australia)	Limestone, moorstone and argillite montane cliffs or shady places under the forest, altitude 100–3300 m.	11	Redefined; Chinese spp. now in *Dorcoceras* and *Damrongia*	Puglisi et al. 2016; Puglisi and Middleton 2018
* Boeica *	Bhutan, S China, N & NE India, Myanmar, N Vietnam, NW Malaya	Shady and damp places and on humid rocks in forests, altitude 200–1400 m.	14	No change	Möller et al. 2017
* Briggsiopsis *	S China (C & S Sichuan, NE Yunnan, Guizhou)	Forests, at stream sides and on rocks in shady places, altitude 250–1500 m.	1	No change	
* Cathayanthe *	S China (Hainan)	Rocks, in wet valleys and ravines; altitude ca. 1800 m.	1	No change	
* Championia *	Sri Lanka	Undisturbed forest, in shady places and loose soil along stream beds.	1	No change	
* Chayamaritia *	Central and eastern Thailand, Laos.	Evergreen and submontane forest in deep shade at 150–1200 m altitude.	2	Genus recently established	Middleton et al. 2015
* Codonoboea *	S Thailand and throughout Malesia, S Japan, E China and Taiwan	Primary forest granite, sandstone and quartz derived soils or rocks.	120	New combination for particular species of *Henckelia* and *Loxocarpus*	Kiew and Lim 2011; Weber et al. 2011a
* Conandron *	E China, Taiwan region of China, S Japan	Humid and wet rocks in forests, altitude 500–1300 m.	1	No change	
* Corallodiscus *	Bhutan, China, N & NE India, Nepal, Thailand	Rocks and rock crevices within forests or above the forest line, from 700 to nearly 5000 m.	5	No change	
* Cyrtandra *	Nicobar Islands and S Thailand through Malesia include Taiwan region of China and the S Pacific to the Hawaiian Islands	Lowland and montane rain forests.	>800	No change	
* Damrongia *	China to Sumatra	Limestone rocks, usually in shade.	11	Re-established for particular species of erstwhile *Chirita*; inclusion of *Boea clarkeana* and the Asian species described in *Streptocarpus*	Weber et al. 2011a; Puglisi et al. 2016
* Deinostigma *	Southern China and Vietnam.	Forests rocks and along trails and roadsides in forested areas; altitude 650–1200 m.	7	Expanded to included several species previously ascribed to *Primulina*	Möller et al. 2016a
* Didissandra *	W Malesia (Sumatra, Malay Peninsula, Borneo, Java)	Lowland and montane rain forests.	8	No change	
* Didymocarpus *	From N and NE India, Nepal and S China southwards to the Malay Peninsula and N Sumatra	Damp (usually acid) rocks or earth banks, in forest or above the forest line, altitude (rarely) sea level to 3500 m.	98	Some spp. transferred to *Petrocodon*	Weber et al, 2011b
* Didymostigma *	SE China (Guangdong, Fujian, Guangxi) & Vietnam	Forests rocks and along trails and roadsides in forested areas; altitude 650–1200 m.	3	No change	
* Dorcoceras *	China, Thailand, Cambodia, Vietnam, Philippines and Indonesia	Shady and damp rocks along trails and roadsides in forests; 100–1500 m.	4	Re-established for particular (non-Australasian) species of *Boea*	Puglisi et al. 2016
* Emarhendia *	Malay Peninsula	Damp limestone rocks, especially at cave entrances.	1	No change	
* Glabrella *	S China, Taiwan region of China	Forests damp rocks and crevices of rocks; 600-1800 m.	3	New genus estblished for 3 spp. of *Briggsia* not to be included in *Oreocharis* or *Loxostigma*	Möller et al. 2014; Wen et al. 2015a,b
* Gyrocheilos *	S China (Guangxi, Guangdong, SE Guizhou), Vietnam	Forests wet places, in valleys and on rocks beside streams; altitude 400–1600m.	5	No change	
* Hemiboea *	C & S China, Taiwan region of China, N Vietnam, S Japan, NE India	Forests and at forest margins rock crevices by streams and wet, shady places in karst regions; altitude 80–2500 m.	34	Inclusion of *Metabriggsia* (2 spp.)	Weber et al. 2011c
* Henckelia *	India, S China, Indo-China Peninsula, Malay Peninsula	Acidic soils and rocks but not on limestone.	~60	Redefined to include *Chirita* p. p. (excl. *Microchirita* and *Primulina*) and *Hemiboepsis*, and to exclude *Codonoboea*	Weber et al. 2011a; Middleton et al. 2013
* Hexatheca *	Borneo (W Kalimantan, Sarawak to Sabah)	Sandstone or limestone rocks.	4	No change	
* Jerdonia *	India (Nilghiri and Anamally Hills)	Rocks in mountains	1	No change	
* Kaisupeea *	Myanmar, Thailand, S Laos	Wet rocks and rocks crevices along streams and waterfalls	3	No change	
* Leptoboea *	Bhutan, N and NE India, S China (Yunnan), Myanmar, Thailand	Hills and mountains	2	No change	
* Liebigia *	Sumatra, Java and Bali	Forest plants, also in disturbed forest, open places and forest margins, river banks etc.; probably growing in acid soil (limestone not recorded, but ecological information generally scanty)	12	Raised from Chirita sect. Liebigia to generic rank	Weber et al. 2011a
* Litostigma *	China (Guizhou, Yunnan)	Wet limestone rocks and at the entrance to caves.	2	Genus recently established	Wei et al. 2010
* Loxocarpus *	S Thailand and E Malesia	Primary forests, often on sloping ground, river banks or on damp rocks.	20	New combinations	Middleton et al. 2013
* Loxostigma *	S China (Sichuan, Yunnan, Guizhou, Guangxi), N Vietnam	Damp, mossy rocks or on tree trunks in forests	11	Recently inclusion of caulescent *Briggsia* species	Möller et al. 2014
* Lysionotus *	From N India and Nepal eastwards through N Thailand, N Vietnam and S China to S Japan	Epiphytically on trees in forest or on damp mossy rocks; 300–3100 m.	29	No change	
* Metapetrocosmea *	S China (Hainan)	Forests and stream sides, altitude 300–700 m.	1	No change	
* Microchirita *	From the Western Ghats of India to the foothills of the Himalayas, through continental SE Asia to Sumatra, Borneo and Java	Wet, light to moderately shady places at cliff bases, on cliff walls in crevices and cracks, or at cave entrances.	37	Raised from Chirita sect. Microchirita to generic rank	Wang et al. 2011; Weber et al. 2011a; Middleton 2018
* Middletonia *	India, Bangladesh, Bhutan, China, Burma, Thailand, Laos, Cambodia, Vietnam, Malaysia	Limestone or granite	5	Genus recently established for four species of *Paraboea*	Puglisi et al. 2016
* Orchadocarpa *	Malay Peninsula (Main Range)	Montane forests, on acid soil.	1	No change	
* Oreocharis *	China, Thailand, Vietnam, Myanmar, Bhutan, NE India, Japan	Shady and damp rocks by streams, in valleys or in forests on slopes or cliffs, dry shaded rocks, altitude 200–3600 m.	>120	Expanded to include *Ancylostemon*, *Bournea*, *Briggsia*, *Dayaoshania*, *Deinocheilos*, *Isometrum*, *Opithandra*, *Paraisometrum*, *Thamnocharis* and *Tremacron*; Inclusion offurther ten spp. of *Briggsia*	Möller et al. 2011b; Möller et al. 2014; Chen et al. 2014
* Ornithoboea *	From S China and Vietnam southwards to N Peninsular Malaysia	Rocks, in shaded, humid places; some (possibly all) species confined to limestone.	16	No change	Scott and Middleton 2014
* Paraboea *	Bhutan, China, Indonesia, Malaysia, Myanmar, Philippines, Thailand, Vietnam	Usually growing on limestone (rarely quartzitic) rocks, in forest or sun-exposed places, altitude 100–3200 m.	141	Expanded by inclusion of *Phylloboea* and *Trisepalum*; removal of four species and placement in the new genus *Middletonia*	Puglisi et al. 2011; Puglisi et al. 2016
* Petrocodon *	China, N Vietnam, NE Thailand	Shady places on rocks cliffs and rocks crevices of limestone hills or in broad-leaved evergreen forests; altitude sea level to 3500 m.	33	Expanded to include *Calcareoboea*, *Didymocarpus*, *Dolicholoma*, *Lagarosolen*, *Paralagarosolen*, *Tengia* and *Wentsaiboea* p. p. (exclude type)	Wang et al. 2011; Weber et al. 2011b
* Petrocosmea *	NE India, S China, Myanmar, Thailand, S Vietnam	Damp rocks and shaded cliffs in forest and above the forest line, altitude 500–3100 m.	49	No change	
* Platystemma *	Nepal, Bhutan, N India, SW China	Shady and damp rocks in valleys or dry cliffs, altitude 2300–3200 m.	1	No change	
* Primulina *	Essentially southern half of China and Vietnam	Limestone	>190	Enormous expansion of the previously monotypic genus by inclusion of Chirita sect. Gibbosaccus, *Chiritopsis*, *Deltocheilos*, and *Wentsaiboea*	Wang et al. 2011; Weber et al. 2011a
* Pseudochirita *	S China (C & W Guangxi), Vietnam	Forests on limestone hills.	1	No change	
* Rachunia *	Thailand, Kanchanaburi province, Thong Pha Phum district, Ban E Tong, near the Thai-Myanmar border at 900 m.	Moist evergreen forest on a slope in shade.	1	Genus recently established	Middleton et al. 2018
* Raphiocarpus *	S China and N & C Vietnam	Montane regions, in shady and damp places under forests, on slopes near streams or in rock crevices.	14	No change since Weber 2004, but changes to be expected	
* Rhabdothamnopsis *	S China	Dense forests, at streamsides in forested areas and in thickets along roadsides, altitude 1600–2200(–4600) m.	1	No change	
* Rhynchotechum *	NE India, Nepal, Bhutan, SW & S China, SE Asia and Malesia to New Guinea	Under broad-leaved forest in valley, shady places near the stream, on the rocks, distributed from coast to 2200 m.	16	No change	
* Ridleyandra *	Malay Peninsula and Borneo	Lowland and (more frequently) montane rain forests.	31	No change	Yunoh and Dzulkafly 2017
* Senyumia *	Malay Peninsula (Pahang: Gunung Senyum and adjacent localities)	Rock faces in damp limestone caves	1	No change	
* Somrania *	Peninsular Thailand	limestone	2	Genus recently established	Middleton and Triboun 2012
* Spelaeanthus *	Malay Peninsula (Pahang), Batu Luas	Damp rock faces, especially at the entrance to limestone caves.	1	No change	
* Tetraphyllum *	NE India, Bangladesh, Myanmar, Thailand	Damp rocks in forest	3	No change	
* Tribounia *	Thailand	Crevices of karst limestone in deciduous forest.	2	Genus recently established	Middleton and Möller 2012
